# Abstract of Proceedings of the Buffalo Medical Association

**Published:** 1868-01

**Authors:** 


ART. III—*Abstract of Proceedings of the Buffalo Medical Association.*

TUESDAY EVENING, December 5, 1867.

The meeting was called to order at the usual hour by the President. Present—Drs. Eastman, Strong, White, Congar, Whitney, Eddy, Rochester, Samo, Potter, Little and Kamerling.

Dr. H. M. Congar was elected Secretary *pro tem.*

DR. WHITE moved that the regular order of business be suspended, and Dr. Hazeltine of Jamestown, Chautauqua county, be invited to participate in the proceedings of the meeting. Carried.

DR. WHITE reported a case of the removal of a foreign body from the rectum of a woman a few days since. The patient had experienced much local pain and tenderness and difficulty of defecation, and had become anæmic and emaciated. Finding no hemorrhids on tactical examination, a foreign body was found, which, on removal, with considerable pain and hemorrhage, proved to be a child's diaper pin, much corroded. The patient was entirely relieved. Nourishing food, tonics and rest, is fast restoring the patient to health.

DR. BOARDMAN reported a case in which a nail two and a half inches long was swallowed by a ruptured child. In a few days the nail was felt in the rupture by the mother, and in due time was passed by the rectum.

Dr. B. also reported a case before reported by him of an adult who, ten or twelve years since, swallowed a twenty dollar gold piece. It was pushed into the stomach at the time. At the end of three or four years the health of the patient was good, but nothing had been heard, seen or felt of the money.

Dr. Strong said he had nothing new to report in the direction suggested by the case just reported; nor did he think it necessary, inasmuch as he believed he was still ahead as to cases of marvellous swallowing and escape, and so might perhaps rest on his laurels. But it may never have been seen by some of the younger gentlemen present, and perhaps is forgotten by others, that some ten years since, more or less, I reported a case of the swallowing of an old-fashioned copper cent by a child of some two and a half years old, and of less than average size and vigor, it being the most delicate of a family of eight children. The cent of the old coinage is pure copper, an inch and two lines in diameter and two lines in thickness. That a metallic body of that size could by any possibility be got into the esophagus of a child so young, seemed so incredible, that for a few days I was in doubt about it, the only evidence being that the child was playing with such a cent, and was seized with a kind of choking, and when asked what he had done with it he pointed to his mouth. I first made a digital examination of the cervical esophagus, and could not find it, and inasmuch as my patient was in no wise suffering, except from occasional retching, I deemed it best to trust unaided nature for the result. This expectant course was followed for four or five days, and the symptoms continuing I introduced a probang, armed with an ivory ball some three-eights of an inch in diameter down to a point near the top of the sternum. Meeting with obstruction at that point I pushed with considerable force down to the stomach. The child could previously swallow fluids without rejecting them, but soon thereafter could take bread with impunity. No symptoms presenting other than occasional vomiting and loss of appetite, I prescribed rest without medication, deeming it quite uncertain if it were even *in* the stomach that it could ever get *out.* But, incredulity notwithstanding, it *did* get out, and after three or four weeks passed per anum. It was impossible to longer doubt for the cent was brought to me for examination.

To my own mind this case is the most astonishing on record of perilous accidental swallowing and escape, when the extreme tenderness of age of the patient and the dimensions of the object swallowed, are taken into account.

*A priori*, I should have said that a metallic substance of that size could neither pass through, *nor be pushed through*, the esophagus of a child two and one-half years old, without rupturing the tube. But the logic of facts is stronger than any—a priori reasoning.

Since this experience I have been entirely composed and at ease, (and this I would inculcate on my younger brethren,) at any ordinary accidental swallowing, such as the nickel cent, the two shilling piece of a former generation, or aught analogous to them in form and dimensions.

On investigation I find the case reported in No. 1 of Vol. xiii of *Buffalo Medical Journal*, to which any one can refer who wishes to read the case more in detail.

Dr. Rochester reported a case of angina pectoris.

Dr. Eastman mentioned the case of premature labor formerly reported to this Association.

Dr. White named a case of extra uterine pregnancy occurring in the practice of Dr. Mack of St. Catharines, C. W., who will soon give a full report of the case to this Association.

On motion, Dr. Henry Nichell was allowed more time to prepare his essay.

Dr. White moved that the essayists hereafter appointed be allowed three months to prepare their essays. Carried.

Dr. Potter was elected to read an essay at the regular meeting in March next.

Dr. Rochester moved that when we adjourn we adjourn for one week, and that the adjourned meeting be for the purpose of acting upon the proposed new fee bill. Carried.

The application of Dr. William C. Phelps for membership was received.

Scarlatina was reported as prevailing.

Adjourned. H. B. Congar, *Sec'y pro tem.*

---

Adjourned meeting held December 10, 1867.

The meeting was called to order at 8 o'clock by the President. Members present—Drs. Eastman, Kamerling, Potter, Trowbridge, Brown, Stone, Wetmore, Cronyn, Samo, Lothrop, Jansen, Smith, Rochester, Boardman and Johnson.

The President stated that the meeting was called for the purpose of discussing and deciding the fee bill.

Dr. Johnson said that he wished to see the fee bill disposed of in some manner, but thought that final action ought to be deferred until a larger number are present, and would therefore move an adjournment.

After considerable talk the motion to adjourn was lost.

Dr. Strong moved that the Secretary read the different items in the proposed bill, and that any member wishing to alter or amend any portion thereof should call attention thereto when read. The motion was carried.

The Secretary then commenced the reading of the bill, which, with few exceptions, was adopted as read.

Dr. Strong moved that members shall, in no case, make out a bill for professional services at a less rate than the minimum established by this fee bill. Carried.

Dr. Rochester moved that the Secretary notify the Insurance Agents of this city that after January 1st, 1868, the fee for a certificate in life insurance, as family physician, will in all cases be five dollars, and chargable to the company for whom the examination is made.

The Secretary was directed by the President to have a sufficient number of the fee bill printed, and in such form as he may think most convenient.

Adjourned. T. M. Johnson, M. D., Sec'y.

